# Synaptic plasticity dysregulation driven by pathogenic tau

**DOI:** 10.1002/alz.087131

**Published:** 2025-01-03

**Authors:** Kristeen A. Pareja‐Navarro, Christina D. King, Grant Kauwe, Doyle Lokitiyakul, Mahima Sharma, Ivy Wong, Yani Ngwala, Birgit Schiling, Tara E. Tracy

**Affiliations:** ^1^ Buck Institute for Research on Aging, Novato, CA USA; ^2^ University of Southern California, Los Angeles, CA USA

## Abstract

**Background:**

Synapses can modify their strength in response to activity, and the unique properties of synapses that regulate their plasticity are essential for memory. Long‐term potentiation (LTP) is considered the physiological basis for how neurons encode new memories. A complex series of postsynaptic signaling events in LTP is associated with memory deficits in tauopathy models, but the mechanism by which pathogenic tau inhibits plasticity at synapses is unknown. Here, our objective was to delineate the postsynaptic mechanisms by which pathogenic tau inhibits LTP in neurons.

**Method:**

We used human induced pluripotent stem cell (iPSC)‐derived neurons with mutations on tau that cause frontotemporal dementia or we treated human iPSC‐derived neurons with tau oligomers to model tau‐related pathogenesis. We induced NMDA receptor‐dependent LTP in the neurons and monitored trafficking of AMPA receptors (AMPARs) to the postsynaptic surface. To identify the mechanisms underlying synapse dysregulation, we designed an approach to detect changes in the postsynaptic proteome with high temporal resolution using APEX‐mediated proximity labeling and mass spectrometry.

**Result:**

Pathogenic tau inhibited the delivery of AMPARs to the postsynaptic surface and blocked LTP expression in human iPSC‐derived neurons. The postsynaptically‐targeted APEX platform mapped the dynamic changes in the postsynaptic proteome of neurons with pathogenic tau.

**Conclusion:**

Our findings reveal new mechanistic insights into how pathogenic tau alters the postsynaptic proteome and inhibits synaptic plasticity.

